# QMachine: commodity supercomputing in web browsers

**DOI:** 10.1186/1471-2105-15-176

**Published:** 2014-06-09

**Authors:** Sean R Wilkinson, Jonas S Almeida

**Affiliations:** 1 Division of Informatics, Department of Pathology, University of Alabama at Birmingham, Birmingham, USA; 2 Department of Biomedical Engineering, University of Alabama at Birmingham, Birmingham, USA

**Keywords:** Cloud computing, Crowdsourcing, Distributed computing, JavaScript, MapReduce, PaaS, Sequence analysis, Web service

## Abstract

**Background:**

Ongoing advancements in cloud computing provide novel opportunities in scientific computing, especially for distributed workflows. Modern web browsers can now be used as high-performance workstations for querying, processing, and visualizing genomics’ “Big Data” from sources like The Cancer Genome Atlas (TCGA) and the International Cancer Genome Consortium (ICGC) without local software installation or configuration. The design of QMachine (QM) was driven by the opportunity to use this pervasive computing model in the context of the Web of Linked Data in Biomedicine.

**Results:**

QM is an open-sourced, publicly available web service that acts as a messaging system for posting tasks and retrieving results over HTTP. The illustrative application described here distributes the analyses of 20 *Streptococcus pneumoniae* genomes for shared suffixes. Because all analytical and data retrieval tasks are executed by volunteer machines, few server resources are required. Any modern web browser can submit those tasks and/or volunteer to execute them without installing any extra plugins or programs. A client library provides high-level distribution templates including MapReduce. This stark departure from the current reliance on expensive server hardware running “download and install” software has already gathered substantial community interest, as QM received more than 2.2 million API calls from 87 countries in 12 months.

**Conclusions:**

QM was found adequate to deliver the sort of scalable bioinformatics solutions that computation- and data-intensive workflows require. Paradoxically, the sandboxed execution of code by web browsers was also found to enable them, as compute nodes, to address critical privacy concerns that characterize biomedical environments.

## Availability

A supporting QM deployment is available at https://v1.qmachine.org, and its source code is available at https://github.com/wilkinson/qmachine. The illustrative examples and their dependencies are provided for live demonstration at http://q.cgr.googlecode.com/hg/index.html along with a screencast and archived genomic data.

## Background

High-performance computing (HPC) for the life sciences is undergoing a fundamental reshaping [1]. The reliance on processor-intensive resources through which ever-enlarging genomics workflows are funneled is giving way to distributed data-intensive infrastructures like TCGA and ICGC [2]. Accordingly, the immovable volumes that are flooding data centers demand “beyond the data deluge” solutions [3] that invert the traditional transfer model so that computations travel to the data and not vice versa. The emphasis, then, is to maximize the availability of the data and the portability of the application. The increasing use of cloud computing infrastructure for biomedical applications reflects the realignment of HPC, as exemplified by the recent partnership between the National Institute for Health (NIH) and Amazon on the 1000 Genomes Project [4].

At the same time, HPC projects such as SETI@home [5], Folding@Home [6], and BOINC [7] have constructed distributed platforms that aggregate commodity hardware and volunteer compute cycles in order to power computationally intensive scientific workflows. In fact, the Folding@Home project currently utilizes the central and/or graphics processing units from more than 250,000 personal computers and video game consoles [8]. To orchestrate concentrated efforts across such large numbers of physical machines and hardware platforms, researchers provide client applications that they must persuade volunteers to download and install permanently on their machines. These applications range in invasiveness from programs that run only when a machine is idle, such as the pioneering SETI@home, to always-on background services like Condor [9] that may tangibly impact a machine’s performance.

The World Wide Web provides a different avenue for HPC, and this is what we explore with QM – a novel direction. The temptation to optimize QM for a particular problem domain was overcome by the greater challenge of creating a system not only to distribute computation across the Web, but also to be “of the Web” itself. A careful study of the Web as a platform reveals that the necessary components are indeed ready for assembly.

The JavaScript (JS) language is not only a “real language” [10] but also a “Lisp in C’s clothing” [11] with support for functional and object-oriented programming styles. Unlike Lisp, however, JS is widely used outside of academia and has ranked among the top twelve most popular languages for more than thirteen years [12]. Scientific libraries in JS are relatively scarce, although a number of specialized libraries such as EBI’s BioJS [13], NIH/NHGRI JBrowse [14], and the recent Genome Maps [15] have emerged to capitalize on the widespread availability of those computational resources, particularly in the genome browsing application domain.

Web browsers execute JS in sandboxed environments that rigorously control access to machine resources, and now those sandboxes implement standardized APIs that provide native capabilities like hardware-accelerated 3D graphics. All modern browsers and even a few browser plugins include just-in-time (JIT) compilers to boost performance [16]. Regular expressions in JS, for example, perform at levels that are no longer matched by Perl [17], the language most often associated with string processing in bioinformatics applications. Moreover, these high-performance JS environments come pre-installed on every personal computer sold today, as well as on smartphones, tablets, gaming consoles, and even televisions. Thus, web browsers represent a modern route for high-performance computing that is well-suited for the “crowdsourcing” model [18]. Indeed, the current fast proliferation of bioinformatics libraries in JS also reflect the advent of web-based “social coding” environments which present entirely novel opportunities for large-scale collaboration [19]. Furthermore, the networking capabilities of the browser platform allow it to import code and data dynamically and thereby orchestrate distributed workflows across multiple browsers on distinct machines, a feature at the core of social computing [20]. Therefore, what is described in this report could be construed as social computing for machines [21], extending the reach of loose distribution models such as mGrid [22].

The emergence of Big Data in the biomedical sciences has been associated with the proliferation of reference databases such as those reviewed yearly by *Nucleic Acids Research*[23]. The aggregation of the Web of Linked Data resources independent of the institutions that host them has been approached by comprehensive data models such as the Distributed Annotation System [24], which we have also explored as a backbone for workflow assembly [25]. It is now amply clear, however, that the linking of data resources, regardless of the domain, is itself domain-neutral and best described by dyadic predicates of W3C’s Resource Description Framework (RDF) that underlies the third generation of Web technologies [21,26,27].

The RDF approach has expanded the basic reliance of unique resource identifiers (URIs) both to identify and locate data (via URLs) which require only a web browser to be put to good use by any researcher, regardless of his expertise or domain of interest. The current extent of its use is dramatically illustrated by the adoption of the RDF framework across all data services of the European Bioinformatics Institute [28]. As also illustrated by some of our work [29-31] developing SPARQL endpoints for TCGA, the volume of the server-side hosted data is not a significant obstacle to developing web applications (“web apps”) that consume those data. On the other hand, mechanisms to assemble workflows for data analysis have not yet matured as user-friendly commodities, despite the availability of excellent frameworks like Taverna [32] and SHARE [33]. One possibility is that the underlying web services themselves need to be amenable to assembly at a moment’s notice – even for deprecated or outdated versions of a procedure. This is an absolute requirement of the modern focus on reproducibility of workflow results [34]. We have explored the use of modular browser-based web apps to deliver this functionality in standard bioinformatics applications such as image analysis [35] and sequence analysis [36]. The success in those two efforts strengthens the claim that script tag loading, the same mechanism web browsers use to load web apps, can orchestrate and distribute the execution of bioinformatics workflows across multiple physical machines. The illustrative, and validating, example detailed in the Results section below will extend the same example of sequence analysis approached in the second of those two reports by analyzing twenty different genomes of *Streptococcus pneumoniae* in parallel.

## Methods

**Figure 1:**
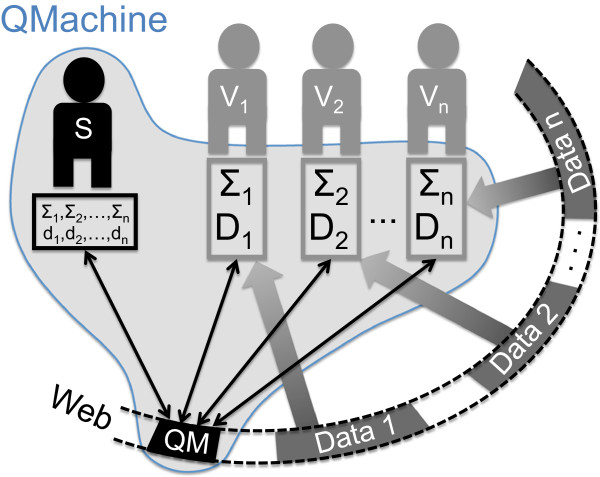
**The abstract architecture.** Architecture of a self-assembled QMachine highlighting the distribution of processing (rectangles) and transfer bandwidth (arrows). QM distributes not only the compute cycles needed to execute the *n* different procedures (*Σ*_1,2,…,*n*_), but also the bandwidth needed the retrieve the corresponding input data (*D*_1,2,…,*n*_) being processed from their respective URLs (*d*_1,2,…,*n*_). The assembly is started by a submitter who issues a key that is endorsed by a number of volunteering web browser sessions (*V*_1,…,*n*_). The code is then transferred to a queue on QM where it is picked up by the volunteering browsers.

QM provides a distributed computing platform as a web service (PaaS). Its architecture (see Figure 1 in Results) combines the general pattern of Web 3.0 technologies with the model used by modern social networking sites by decoupling the presentation/analytical layer from the persistent representation layer so that the former runs on the client side as a web app that consumes an application programming interface (API) presented by the latter on the server side. QM also decouples the presentation and analytical layers of the web app so that third parties may embed the QM web service as part of their own web apps.

To provide this functionality, QM contains three main components: an API server, a web server, and a website. The API and web servers are written completely in JS, and the website is written in HTML5, CSS, and JS. Nothing about QM’s design or interface binds it to a particular development stack, but our desire to construct the project as a true Web Computing “device” motivated us to implement as much of the code in JS as possible. The strategy paid unexpected dividends, as well; the server-side components are free from assumptions about the hardware and operating systems on which they run, which vastly simplifies deployment to the cloud via Platform-as-a-Service (PaaS) [37].

This report also presents code examples (see Results) which can be run from any website that embeds the QM web service. The examples are all written in JS, but some of them also make use of CoffeeScript, “a little language that compiles into JavaScript” [38]. Many common scientific languages can be translated to and from JS, and a comprehensive list of projects for this purpose is available at http://bit.ly/altjsorg..

### API server

The API server is a program which responds to requests sent by clients over the standard Hypertext Transfer Protocol (HTTP). The program then interprets the requests according to their methods, target URLs, and embedded data. QM’s API presents three operations, as shown in Table 1. Data sent as part of a POST should be formatted in JavaScript Object Notation (JSON) format; response data from QM are JSON-formatted, too. Note that clients need not be browsers – any software package that can communicate over HTTP and manipulate JSON-formatted data can use QM directly.

**Table 1:** HTTP API

**HTTP request**			**HTTP response**	
**Method**	**Example URL**	**Data**	**Code**	**Data**
GET	/box/sean?key=some_job_id		200	{ }
GET	/box/sean?status=waiting		200	[ ]
POST	/box/sean?key=some_job_id	{ }	201	

This table specifies the Application Programming Interface (API) understood by QM’s API server. Request and response data use JavaScript Object Notation (JSON) format, but data may be omitted where values are left blank. The “**{ }**” denotes a JSON object, and the “**[ ]**” denotes a JSON array.

The API server is implemented as a simple Node.js [39] program that loads and executes all of its application logic from QM’s own publicly available module, “qm”, using the Node Package Manager (NPM) [40]. The module supports five different databases as targets for persistent storage: Apache CouchDB [41], MongoDB [42], PostgreSQL [43], Redis [44], and SQLite [45]. These five open-source databases were chosen for support based on their high performance and popularity, and their differences in design help to guide the development of QM as an HPC solution for a heterogeneous database landscape. The relative merits of the alternative implementations to the default use of MongoDB are as follows. CouchDB and MongoDB are both document-centric NoSQL databases with MapReduce APIs that understand JS, but their designs are very different. CouchDB is more than just a database – it is nearly sufficient to implement QM by itself because it bundles a web server and an HTTP-accessible API. MongoDB, by way of contrast, has an API that mimics the traditional relational style used by PostgreSQL and SQLite, with a much stronger focus on clustering and “sharding” (horizontal partitioning) across nodes. PostgreSQL represents relational database management systems (RDBMS), the workhorses that traditionally power enterprise applications and data warehouses, while SQLite represents embedded (serverless) databases. Redis is an in-memory key-value store that is often referred to as a “data structure server” because its keys can contain strings, hashes, lists, sets, and sorted sets. The ability to map QM’s persistent representation layer across such a wide variety of storage systems simplifies deployment and maintenance significantly. The service that backs this report’s illustrative examples, available at https://v1.qmachine.org, uses MongoDB.

QM’s API server supports Cross-Origin Resource Sharing (CORS) [46] so that any webpage can embed QM to distribute workflows across web browsers without violating the Same-Origin Policy [47]. There is wide support for CORS in web browsers [48].

### Web server

The web server, like the API server, is implemented as a Node.js program, and its logic is contained in the same NPM module, “qm”. That is, the installation of all of QMachine’s base libraries can be achieved simply by running Node’s built-in module management system: npm install qm. It is worth recalling the minimal role played by the server-side components of QM (see Figure 1 in Results). The web server exists only to provide the presentation/analytical layer’s resources to client machines. Because these resources are static, the web server can be replaced by off-the-shelf web servers like Apache [49] and Nginx [50].

### Website

The website functions as the presentation/analytical layer of QM. It was developed as a browser client for the QM API, and thus it is implemented in HTML5, CSS, and JS, as can be verified by inspecting its source code at https://github.com/wilkinson/qmachine. The website consists of a single webpage that communicates with the API server periodically via XMLHttpRequest (XHR) using a technique known as Asynchronous JavaScript and XML (AJAX). The “AJAX” name is a bit misleading, however, because XHR is not limited to handling XML data; all of the browser client’s communications with the API server use JavaScript Object Notation (JSON).

When a browser loads the webpage, it initially loads only the presentation layer, comprised of the HTML, CSS, and JS resources necessary to render the graphical user interface (GUI). Immediately after the GUI loads, the browser retrieves QM’s analytical layer, which is written entirely in JS. This design improves the user experience by loading the GUI faster, and it isolates the presentation layer’s code from the analytical layer’s code. Thus, third parties can embed QM’s analytical layer and thereby use QM’s persistent representation layer without loading QM’s presentation layer, as shown by the examples at https://v1.qmachine.org/barebones.html and http://q.cgr.googlecode.com/hg/index.html.

QM’s browser client models a workflow as a set of transforms that should be applied to input data in a specific order to produce output data. A “task description” is an object that contains the transform *f*, the data *x*, and any information needed to prepare the environment prior to execution.

As described above, the client-side application that is distributed when a browser is pointed to https://v1.qmachine.org was developed using only web technologies: HTML5, JS, and CSS. In order to stay within the core JS syntax that is supported by all browsers and all platforms – including mobile devices – code development was assisted by JSLint [51]. JSLint is also used directly within the analytical layer as a static analysis tool to identify tasks which can be serialized faithfully into JSON for distribution to volunteer machines. A generic library, Quanah [52], was also developed to solve the numerous concurrency challenges faced in asynchronous data transfer by QM; it is therefore a key component of the prototype described here and is accordingly also made publicly available with open source. The presentation layer uses jQuery [53] and Twitter Bootstrap [54] to ensure consistent look-and-feel across a variety of mobile and desktop browsers. The GUI additionally attempts to support outdated browsers through optional integration with Google Chrome Frame [55], HTML5 Shiv [56], and json2.js [57], but it does so only as a courtesy.

### Demonstration program

The demonstration program is written in pure JavaScript so that it can be run in an ordinary web browser without dependencies on any native applications, plugins, or add-ons. It extends a demonstration from a previous study [36] in which a MapReduce decomposition of a sequence analysis procedure was used to find the longest similar segment between a user-given sequence and a full bacterial genome. The demonstration in this study will not only reproduce the previous results using remote execution on another machine, but it will do so in parallel for all of the twenty strains of *Streptococcus pneumoniae* that are currently available from the National Center for Biotechnology Information (NCBI). It uses an updated version of the same Universal Sequence Maps (USM) [58] library used by the previous study, as referenced directly by exact version from its online repository.

## Results

The architecture of a QMachine detailed in Figure 1 follows the general pattern of Web 3.0 technologies by using the server side exclusively for persistent representation and leaving the rest of the program logic to run on the client side. QM uses a key-value architecture to orchestrate volunteering client machines in a manner that maximizes the distribution of the computational resources required for data transfer and subsequent data processing. This orchestration is highlighted in Figure 1: QM distributes not only the compute cycles needed to execute the *n* different procedures (*Σ*_1,2,…,*n*_), but also the bandwidth needed the retrieve the corresponding input data (*D*_1,2,…,*n*_) being processed from their respective URLs (*d*_1,2,…,*n*_). This design is motivated by the constraints of biological applications such as next generation sequencing in which the limiting factor is more often the available memory than the processor speed.

**Figure 2:**
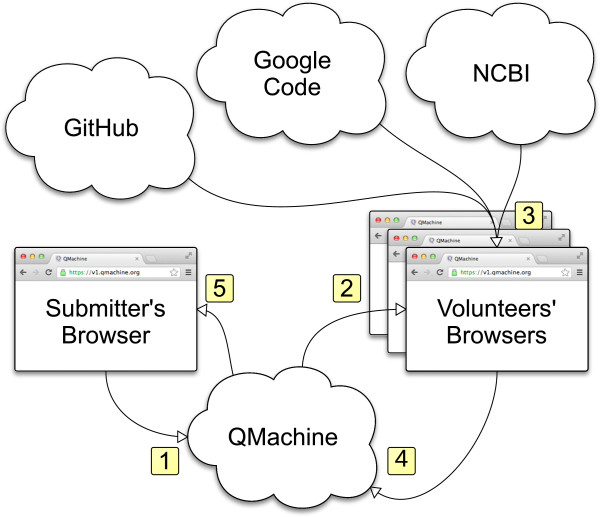
**A workflow for real-world genomic analysis. **(1) The submitter interactively calls the high-level QM.map function from a web browser with the URLs of twenty different *Streptococcus pneumoniae* genomes as input, causing the client to submit twenty individual task descriptions to QM’s API server. (2) A volunteer’s browser, polling for new task descriptions on QM’s API server, finds and downloads a task description. (3) The volunteer’s browser executes the task after downloading three external resources: the USM library served from GitHub, the JMat library served from Google Code, and the bacterial genome served from NCBI. (4) The volunteer’s browser returns the results of the task execution to QM’s API server and resumes polling for new task descriptions. (5) The submitter’s browser, polling for updates to the individual task descriptions, retrieves the results from QM’s API server.

The operation of QM relies on the creation of unique identifiers to define “boxes” that are then shared with the volunteering browsers in a manner resembling traditional API keys. This operation will be described in a series of four examples that increase in complexity, beginning with (1) the remote execution of a simple algebraic operation, followed by (2) distribution of the same operation as a parallel (map) transformation of the elements of an array and (3) distribution again as part of a MapReduce procedure; finally, the (4) parallel execution of a real-world genomic sequence analysis in which both the code and the data needed to perform the analysis are invoked by a single submitter but then entirely resolved and executed asynchronously by multiple volunteer browsers. The final, real-world example distributes both the processing and networking loads, as described in Figure 2. It illustrates the ability of volunteer nodes to call code and data from multiple sources which are independently developed and maintained. This illustrative series is also available as a YouTube webcast at http://goo.gl/tnpMiQ.

### Loading the client-side library

QM’s analytical layer is provided by a JS library that can be loaded by any web browser automatically as part of any webpage that contains the following code: 

 Once loaded, the JS environment will contain a global object named QM with convenient high-level methods that can be used to reproduce the results of the four examples that follow.

#### ***(1) Simple algebraic operation***

For the first illustrative example, let *f* be a function that increments a given number *x *by 2, and let *x *= 2. To compute the result, *f*(*x*), on a volunteer machine, we could use the QM.submit method: 

 As in the rest of the illustrative series, this example is described and demonstrated in the accompanying screencast (http://goo.gl/tnpMiQ). Note also that this simple operation is easily expressed in other languages such as CoffeeScript [38]): 

 As discussed in “Methods”, QM’s architecture does not impose the use of a specific programming language, as long as a compiler to JS, the web’s “assembler language” [35], is distributed with the remote call. To support this claim, the QM client library delegates to a compiler – written in JS – for the CoffeeScript language. For a list of compilers that can translate programs written in other languages into JS so that they can be interpreted by volunteering browsers, see http://bit.ly/altjsorg.

#### ***(2) Simple distributed map***

Because each QM.submit operation is an asynchronous call, multiple calls can run simultaneously. Thus, it is straightforward to distribute the execution of a “map” function, a higher-order functional pattern that applies the same operation to each element of an array. This pattern is so ubiquitous in scientific computing that it warrants a dedicated method, QM.map, that can be used as follows: 

#### ***(3) Simple distributed MapReduce***

Just as in the “map” function shown above, it is straightforward to distribute the execution of a “reduce” function, a higher-order functional pattern which combines elements of an array two at a time until only one value remains. As recently surveyed by Zou et al. [59], the MapReduce programming template is at the very core of modern computationally intensive bioinformatics applications. This third illustration demonstrates the MapReduce pattern as an extension of the second example by subsequently summing the results of the distributed “map” using a “reduce” also distributes across QM’s volunteers: 

#### ***(4) Real-world genomic analysis***

The fourth illustrative example assesses QM’s ability to scale the asynchronous operations demonstrated above for use in a real-world bioinformatics workflow. The example is a Fractal MapReduce decomposition of sequence alignment [36] which distributes both the processing and networking loads across QM’s volunteers, as described in Figure 1. It also demonstrates that libraries of any complexity or elaboration can be distributed to the volunteers along with the commands that invoke those libraries. Specifically, both the data and the library encoding for the sequence analysis procedure are invoked by QM but entirely resolved and executed by the volunteer browsers. It also illustrates the ability of a volunteer node to call code and data from multiple sources which are independently developed and maintained.

Consider, as in the first example, that we have some *x* that we wish to transform via some function *f*, so that x is now an array of URLs that reference FASTA files hosted by NCBI: 

 We want to perform a particular sequence analysis on each FASTA file, namely a Fractal MapReduce decomposition of the Chaos Game Representation [36]. Thus, we define a function f for use with the QM.map method that will take a URL as input and return the results of the sequence analysis as output: 

 There is a key challenge, however, in that our function f depends on a usm function that exists only after an external library has been loaded. Therefore, to specify the task completely, we will need either to include usm as part of f or else to pass a reference to the library in the form of a URL. We chose the latter strategy in this case so that the library can be downloaded in parallel by each volunteer simultaneously without burdening the API server. Each external function may have multiple dependencies, and thus QM.map accepts an optional env parameter so that the dependencies for each external function can be specified as an array of URLs to be loaded sequentially: 

 Finally, we will specify the box parameter explicitly for demonstration purposes. The box parameter takes the place of an API key and allows volunteers to execute tasks in a particular queue. This mechanism allows submitters to direct tasks into different queues and further enables the use of abstractions like MapReduce: 

 Putting these definitions together, we now launch twenty individual genomic sequence analyses for simultaneous execution via 

 A full version of these examples can be found online at http://q.cgr.googlecode.com/hg/index.html. The version there includes the full URLs to all twenty *Streptococcus pneumoniae* genomes and also to the versioned libraries specified by env. An accompanying screencast for these examples is also provided in that page.

### Usage statistics

**Figure 3:**
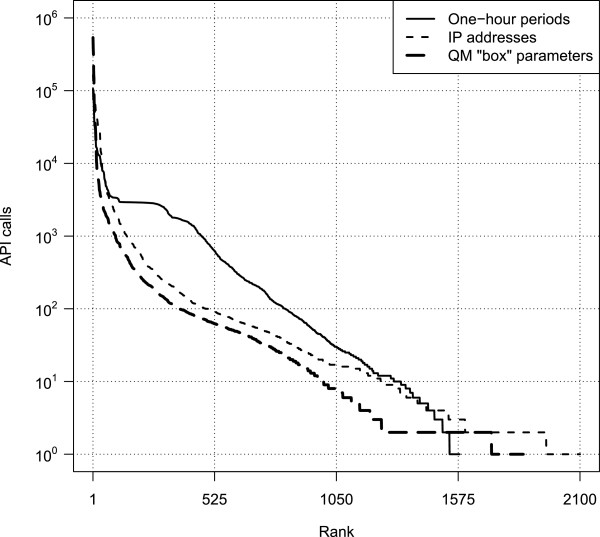
**Three representations of usage data. **This plot illustrates worldwide usage of the QMachine web service from log data collected from April 2013 to April 2014. More than 2.2 million calls were made to its Application Programming Interface (API), the details for which are shown in Table 1. The thin, solid curve represents the number of calls made to its Application Programming Interface (API) server by hour; QM was idle (no calls received) during approximately 79% of all one-hour periods, and those hours were omitted. The thin, dashed line represents the number of API calls aggregated by IP address. The thick, dashed line represents the number of API calls made to a particular “box” on QM; see the Results section for further details about QM’s boxes.

**Figure 4:**
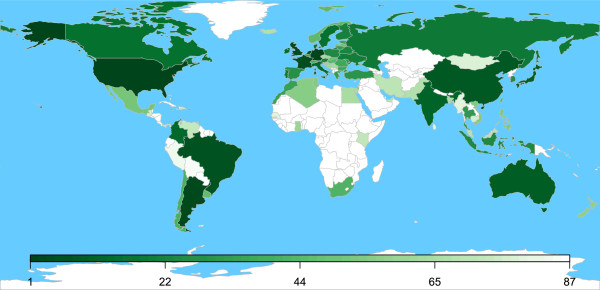
**Geographical distribution of API calls.** This illustrates the usage of the QMachine web service by country as a world map. More than 2.2 million API calls were received from visitors in 87 countries, as identified by IP address. Each country’s shade of green varies from pale to dark in relation to its rank as sorted from greatest to least.

The dissemination of browser-based tools in social coding environments like GitHub [19] is characterized by the same expansive dynamics as social media. For example, although this is our first report describing it, QM can be – and has been – discovered by the community at large. During the 12 months period beginning in April 2013, QM received more than 2.2 million API calls from 2,100 IP addresses in 87 countries to more than 1,800 QM “boxes” (the code and results exchange domains defined by token), with 98 boxes receiving more than 1,000 calls each and 16 boxes receiving 10,000 calls or more. The statistics of QM usage are described in Figure 3, and the geographic distribution of its users is described in Figure 4. It is unclear exactly how much of QM’s usage is associated with the distributed computational genomics web apps that motivated its development, but the wide geographic distribution of its users suggests an appeal driven by a more general interest in distributed computing. This interpretation is reinforced by unsolicited reports about QM in HPC media such as *HPCwire* (article at http://goo.gl/9H5W03) and *insideHPC* (http://goo.gl/bDkJZL). Finally, as noted in Methods, all of the server- and client-side software are open-source and permissively licensed. The browser client requires nothing more than script tag loading to be included in a web app, and the server is just as accessible through NPM [40]. It is therefore conceivable that other QM deployments are in use at other addresses, perhaps even within the firewall of medical centers, as was the specific intention of QM’s development.

The server load associated with orchestrating this initial heavy use of QM is very modest because of the reliance on code distribution rather than on code execution. In fact, the deployment supporting the usage statistics described above (the server behind https://api.qmachine.org) was never overwhelmed by traffic spikes even though it was running on a shared-tenant virtual machine with just 512 MB of RAM, 2×512 MB MongoDB databases, and no hard drive. Furthermore, the authors do not incur any maintenance costs for the public tool dissemination, either from GitHub or from NPM’s package repository. We are therefore committed not to collect any data beyond the broad statistics described in Figures 3 and 4 for the reference deployment discussed here. Particularly relevant for the biomedical usage scenario that motivated this work, we are also committed not to collect any data at all from private deployments of QM; in other words, no part of QM’s software ever sends data back to our server(s) from other deployments. This design allows administrators to deploy their own QM servers through NPM and fully configure their own security as needed for clinical and/or biomedical research usage. These assurances can, of course, be verified through inspection of QM’s source code.

## Discussion

**Figure 5:**
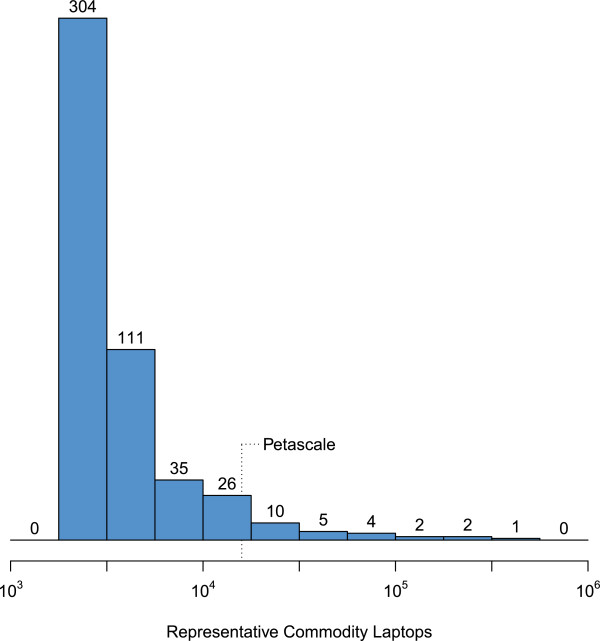
**Performance distribution of the “Top500” supercomputers.** This histogram shows the floating-point performance distribution of the “Top500” fastest supercomputers, given in terms of representative commodity laptops. The data used were taken from the list published in November 2013 at http://top500.org and compared to the results obtained by running the same LINPACK benchmark [60] on a commodity laptop (Core i7-2720QM, 8GB 1333MHz DDR3 SDRAM). Simple division of the real-world performance yielded the estimated performance as multiples of the commodity laptop.

QMachine is a web service for executing distributed workflows that can use ordinary web browsers as the ephemeral compute nodes of a crowdsourced supercomputer. The idea here is simple: commodity computers equipped with web browsers join an abstract machine by visiting a website, and they unjoin by navigating to a different site or by closing the browser. While a browser remains on the site, it reacts to input from the user and from the site’s backend infrastructure by executing JS, which provides the abstract machine with some potential to perform computations. At any instant, the net computational potential available to a high-traffic website falls well within the HPC range, as shown in Figure 5. QM enables this potential to be harnessed with no nominal cost through volunteer computing.

### MapReduce

Many researchers with access to large-scale computational resources still find those resources inaccessible because “everyday” workflows often require more than just fast computers – they require programming skills that are harder to acquire. Bioinformatics workflows increasingly rely on MapReduce as an abstraction, but available MapReduce resources still expose researchers to programming environments with strict procedural requirements and steep learning curves. QM is much simpler to set up and operate than Apache Hadoop [61], for example. It allows users to run MapReduce jobs on multiple physical machines and to crowdsource elastic computing resources with the simplicity of writing and loading a webpage – skills performed every day by millions of people worldwide. We argue that using the web computing architecture explored by QM – that is, without installing a dedicated application – is a natural evolution of current cloud-based MapReduce services, just as Hadoop was a step up from one-off compile-and-run workflows.

### Distributed computing

QM’s web service provides a message passing interface for distributed computing. This statement may at first sound paradoxical, but JS’s single-threaded programming model does not limit JS programs to single-threaded execution; external execution contexts can be used to support concurrency via event-driven programming. QM leverages browsers’ asynchronous (non-blocking) network communications layers to connect multiple machines’ execution contexts, but browsers that support Web Workers [62] can execute concurrent programs within the same physical machine.

### Cloud browsers

An interesting new twist in the development of web computing architectures is the emergence of the “cloud browser” [63]. In these systems, a mobile browser behaves as a thin client when a webpage’s scripts demand heavy computation. Cloud browsers therefore demonstrate browser scaling in the vertical direction, whereas QM demonstrates browser scaling in the horizontal direction. Because QM makes no assumptions about its volunteers’ underlying resources, cloud browsers can volunteer for QM alongside ordinary browsers without loss of generality. In other words, cloud browsers represent enhancements of present-day browsers, while QM presents a solution for HPC that advances the underlying architecture of the Web towards that of a Global Computer [64,65].

### Biomedical applications

In clinical environments, it can be difficult or even impossible to distribute workflows due to privacy concerns that prevent sensitive data from leaving the hospital environment, where conventional HPC is typically absent. QM satisfies this preoccupation without requiring additional resources. As shown in Figure 5, the median computational power of the Top500 HPC in November 2013 (http://goo.gl/XIUIDP) was roughly 2,600 times faster than our lab’s standard-issue desktop machine. This is a much smaller factor than the number of machines in a typical medical center. Thus, even if restricted to a single hospital environment, volunteer computing can still rival the total capacity of very substantial HPC resources.

QM can also be used to power workflows inside of a single workstation. In such a scenario, the workstation would run QM’s API server locally and use multiple browser tabs to execute the workflow in parallel. Such a workflow might also incorporate existing bioinformatics tools such as the Basic Local Alignment Search Tool (BLAST) [66] by using traditional server-side scripting languages like Perl [67] or Python [68] to connect to QM’s API or even directly to the persistent storage layer.

### Security

The security of workflows that use QM is handled orthogonally to QM by the selection of volunteers and by access control to code and data. A number of considerations should nevertheless be made to assist in the configuration of its distributed operation. It is important to recall that the web browser executes JS within a sandboxed environment, which, among other protections, prevents programmatic access to the filesystem of the volunteer machine. As a result, QM’s security is configured around two firewalls.

The first and most basic protection is associated with the uniqueness of the “box” (token) issued by the submitter, which should be shared only with trusted volunteers. An additional layer of security can be added through the use of open authentication such as OAuth 2.0 [69] to verify that only trusted volunteers are involved. This second layer of protection is particularly useful in creating audit trails. These two mechanisms can be combined in many ways, as appropriate for a particular workflow. For example, different steps of a workflow could be assigned to distinct cohorts of volunteers depending on the sensitivity of the code and data and/or the trustworthiness of the volunteers. The resulting granularity could also be used to build redundancy – and therefore robustness – into the distributed QM operation.

In short, the weakest link in QM’s architecture – and where the opportunities for abuse lie – derive from the sharing of the “box” by members of a group of volunteers. In this regard, the key feature of QM’s design is that the abuse can target the submitters but not the volunteers, because QM’s operations take place within the sandbox of the web browser.

## Conclusions

QMachine was developed to respond to the challenges of – and to capitalize on the opportunities of – bioinformatics applications encountered in biomedical environments. For more than a decade, volunteer computing has enticed computational biology as a scalable and cost-effective high-performance computing solution. QM essentially ports that solution to the modern computational landscape, which is increasingly dominated by mobile hardware platforms and the use of the web browser as the universal software platform. The features of the modern web browser go beyond those that make it a high-performance computational environment with advanced communication layers; they also include the transformative feature that computations run in a robust sandbox that prevents access to the underlying machine’s potentially sensitive filesystem. QM also responds to another modern trend towards engaging HPC resources through the use of the MapReduce programming pattern, rather than through direct interactions with compute nodes. The sequence analysis application that illustrates the use of QM in this report offers the sort of immediate utility that would benefit bioinformatics applications in Medical Genomics. It is argued, however, that QM, as an “of the Web” distributed computing system, may be just as useful in the identification of the fundamental features of pervasive web computing.

## Availability of supporting data

The *Streptococcus pneumoniae* genome data are used directly from the publicly available online repository at http://ftp.ncbi.nlm.nih.gov/genomes/Bacteria/, and the relevant FASTA files have also been archived to http://q.cgr.googlecode.com/hg/data/, a version-controlled repository. The original data used to produce Figure 5 were taken from http://s.top500.org/static/lists/xml/TOP500_201311_all.xml and are archived to http://q.cgr.googlecode.com/hg/data/.

### Source code

All source code for this paper is version-controlled and open-sourced. The primary source for QMachine’s code is located in a Git [70] repository at https://github.com/wilkinson/qmachine. The code and data for the illustrative examples shown in the Results section are available in a Mercurial [71] repository at http://q.cgr.googlecode.com/hg/. Quanah’s source code repository is available at https://github.com/wilkinson/quanah, and the USM repository is available at https://github.com/usm/usm.github.com.

## Competing interests

The authors declare that they have no competing interests.

## Authors’ contributions

The original concept is due to JSA, and both authors made substantial contributions to the design interfaces of QM. SRW designed Quanah and implemented both QM and Quanah, and JSA designed and implemented USM. Both authors developed the report and tested the illustrative examples. Both authors read and approved the final manuscript.

## References

[B1] Schadt EE Linderman MD Sorenson J Lee L Nolan GP **Computational solutions to large-scale data management and analysis** Nat Rev Genet 2010 11 647 657 10.1038/nrg2857 20717155 PMC3124937

[B2] Ledford H **Big science: the cancer genome challenge** Nature 2010 464 7291 972 974 [http://dx.doi.org/10.1038/464972a] 10.1038/464972a 20393534

[B3] Bell G Hey T Szalay A **Beyond the data deluge** Science 2009 323 1297 1298 10.1126/science.1170411 19265007

[B4] Cravedi K Randall T Thompson L **1000 genomes project data available on amazon cloud** 2012 [http://www.genome.gov/27548042]

[B5] Anderson D Werthimer D Cobb J Korpela E Lebofsky M Gedye D Sullivan WT Lemarchand G, Meech K **SETI@home: internet distributed computing for SETI** Bioastronomy 99, Volume 213 of Astronomical Society of the Pacific Conference Series 2000 San Francisco: Astronomical Society of the Pacific 511 511

[B6] Shirts MR **Pande VS: Screen savers of the world, unite!** Science 2000 314 1903 1904 17742054 10.1126/science.290.5498.1903

[B7] Anderson DP **BOINC: a system for public-resource computing and storage** Proceedings of the 5th IEEE/ACM International Workshop on Grid Computing, GRID ‘04 2004 Washington, DC: IEEE Computer Society 4 10 [http://dx.doi.org/10.1109/GRID.2004.14]

[B8] **Folding@Home Project Statistics** [http://fah-web.stanford.edu/cgi-bin/main.py?qtype=osstats]

[B9] Thain D Tannenbaum T Livny M **Distributed computing in practice: the condor experience** Concurrency Pract Ex 2005 17 2–4 323 356

[B10] Mikkonen T **Taivalsaari A: Using JavaScript as a real programming language** Tech. rep., Sun Microsystems, Inc., Mountain View, CA, USA 2007

[B11] Crockford D JavaScript: The Good Parts 2007 Sebastopol: O’Reilly

[B12] **TIOBE Index** [http://www.tiobe.com/index.php/content/paperinfo/tpci/index.html]

[B13] Gómez J Garcia LJ Salazar GA Villaveces JM Gore SP Castro AG Martin MJ Launay G Alcántara R del Toro N Dumousseau M Orchard SE Velankar S Hermjakob H Zong C Ping P Corpas M Jimenez RC **BioJS: an open source JavaScript framework for biological data visualization** Bioinformatics 2013 29 8 1103 1104 [http://dblp.uni-trier.de/db/journals/bioinformatics/bioinformatics29.html\#GomezGSVGGMLAdDOVHZPCJ13] 10.1093/bioinformatics/btt100 23435069 PMC3624812

[B14] Westesson O Skinner M Holmes I **Visualizing next-generation sequencing data with JBrowse** Brief Bioinform 2013 14 2 172 177 [http://bib.oxfordjournals.org/content/14/2/172.abstract] 10.1093/bib/bbr078 22411711 PMC3603211

[B15] Medina I Salavert F Sanchez R de Maria A Alonso R Escobar P Bleda M Dopazo J **Genome maps, a new generation genome browser** Nucleic Acids Res 2013 41 W1 W41 W46 [http://nar.oxfordjournals.org/content/41/W1/W41.abstract] 10.1093/nar/gkt530 23748955 PMC3692043

[B16] Rohlf C Ivnitskiy Y **The security challenges of client-side just-in-time engines** IEEE Secur Privacy 2012 10 2 84 86 [http://doi.ieeecomputersociety.org/10.1109/MSP.2012.53]

[B17] **The Computer Languages Benchmarks Game** [http://benchmarksgame.alioth.debian.org]

[B18] Sansom C **The power of many** Nat Biotechnol 2011 29 201 203 10.1038/nbt.1792 21390017

[B19] Dabbish L Stuart C Tsay J Herbsleb J **Social coding in GitHub: transparency and collaboration in an open software repository** Proceedings of the ACM 2012 conference on Computer Supported Cooperative Work, CSCW ‘12 2012 New York: ACM 1277 1286 [http://doi.acm.org/10.1145/2145204.2145396]

[B20] Eysenbach G **Medicine 2.0: social networking, collaboration, participation, apomediation, and openness** J Med Internet Res 2008 10 e22 10.2196/jmir.1030 18725354 PMC2626430

[B21] Berners-Lee T Hendler J **From the semantic web to social machines: a research challenge for ai on the world wide web** Artif Intell 2010 174 156 161 10.1016/j.artint.2009.11.010

[B22] Karpievitch Y Almeida J **mGrid: A load-balanced distributed computing environment for the remote execution of the user-defined Matlab code** BMC Bioinformatics 2006 7 139 [http://www.biomedcentral.com/1471-2105/7/139] 10.1186/1471-2105-7-139 16539707 PMC1431572

[B23] Galperin MY Fernandez-Suarez XM **The nucleic acids research database issue and the online molecular biology database collection** Nucleic Acids Res 2012 **40** 10.1093/nar/gkr1196 PMC3245068 22144685

[B24] Prlic A Down TA Kulesha E Finn RD Kahari A Hubbard TJ **Integrating sequence and structural biology with DAS** BMC Bioinformatics 2007 8 333 10.1186/1471-2105-8-333 17850653 PMC2031907

[B25] Veiga DFT Deus HF Akdemir C Vasconcelos ATR Almeida JS **DASMiner: discovering and integrating data from DAS sources** BMC Syst Biol 2009 3 109 10.1186/1752-0509-3-109 19919683 PMC2789070

[B26] Hendler J **Web 3.0 emerging** Computer 2009 42 111 113

[B27] Hendler J Holm J Musialek C Thomas G **US government linked open data: Semantic.Data.Gov** IEEE Intell Syst 2012 27 25 31

[B28] Jupp S Malone J Bolleman J Brandizi M Davies M Garcia L Gaulton A Gehant S Laibe C Redaschi N Wimalaratne SM Martin M Le Novère N Parkinson H Birney E Jenkinson AM **The EBI RDF Platform: linked open data for the life sciences** Bioinformatics 2014 30 9 1338 1339 10.1093/bioinformatics/btt765 24413672 PMC3998127

[B29] Deus HF Veiga DF Freire PR Weinstein JN Mills GB Almeida JS **Exposing the cancer genome atlas as a SPARQL endpoint** J Biomed Inform 2010 43 6 998 1008 [http://www.sciencedirect.com/science/article/pii/S153204641000136X] 10.1016/j.jbi.2010.09.004 20851208 PMC3071752

[B30] Robbins DE Grüneberg A Deus HF Tanik MM Almeida JS **A self-updating road map of the cancer genome atlas** Bioinformatics 2013 [http://bioinformatics.oxfordjournals.org/content/early/2013/04/17/bioinformatics.btt141.abstract] 10.1093/bioinformatics/btt141 PMC3654710 23595662

[B31] Saleem M Padmanabhuni SS Ngomo ACN Almeida JS Decker S Deus HF **Linked cancer genome atlas database** Proceedings of the 9th International Conference on Semantic Systems, I-SEMANTICS ‘13 2013 New York: ACM 129 134 [http://doi.acm.org/10.1145/2506182.2506200]

[B32] Hull D Wolstencroft K Stevens R Goble C Pocock M Li P Oinn T **Taverna: a tool for building and running workflows of services** Nucleic Acids Res 2006 34 729 732 10.1093/nar/gkl320 PMC1538887 16845108

[B33] Vandervalk BP Mccarthy EL Wilkinson MD **SHARE: a semantic web query engine for bioinformatics** Proceedings of the 4th Asian Conference on The Semantic Web, ASWC ‘09 2009 Berlin, Heidelberg: Springer-Verlag 367 369 [http://dx.doi.org/10.1007/978-3-642-10871-6_27]

[B34] Peng RD **Reproducible research in computational science** Science 2011 334 1226 1227 10.1126/science.1213847 22144613 PMC3383002

[B35] Almeida JS Iriabho E Gorrepati VL Wilkinson S Robbins DE Gruneberg A Hackney JR **ImageJS: personalized, participated, pervasive and reproducible image bioinformatics in the web browser** J Pathol Informat 2012 3 25 10.4103/2153-3539.98813 PMC3424663 22934238

[B36] Almeida JS Gruneberg A Maass W Vinga S **Fractal MapReduce decomposition of sequence alignment** Algorithm Mol Biol 2012 7 12 10.1186/1748-7188-7-12 PMC3394223 22551205

[B37] Lenk A Klems M Nimis J Tai S Sandholm T **What’s inside the Cloud? An architectural map of the cloud landscape** Proceedings of the 2009 ICSE Workshop on Software Engineering Challenges of Cloud Computing, CLOUD ‘09 2009 Washington, DC: IEEE Computer Society 23 31 [http://dx.doi.org/10.1109/CLOUD.2009.5071529]

[B38] **CoffeeScript** [http://coffeescript.org/]

[B39] **Node.js** [http://nodejs.org/]

[B40] **Node Package Manager** [https://npmjs.org/]

[B41] **Apache CouchDB** [https://couchdb.apache.org/]

[B42] **MongoDB** [http://www.mongodb.org/]

[B43] **PostgreSQL** [http://www.postgresql.org/]

[B44] **Redis** [http://redis.io/]

[B45] **SQLite** [https://www.sqlite.org/]

[B46] **Cross-Origin Resource Sharing** [http://www.w3.org/TR/cors/]

[B47] **Same-Origin Policy for JavaScript** [https://developer.mozilla.org/en/Same\_origin\_policy\_for\_JavaScript]

[B48] **Can I use CORS?** [http://caniuse.com/#feat=cors]

[B49] **The Apache HTTP Server Project** [https://httpd.apache.org/]

[B50] **Nginx** [http://nginx.com/]

[B51] **JSLint** [http://www.jslint.com/]

[B52] **Quanah** [http://wilkinson.github.com/quanah/]

[B53] **jQuery** [http://jquery.com]

[B54] **Twitter Bootstrap** [http://twitter.github.com/bootstrap/]

[B55] **Google Chrome Frame** [https://www.google.com/chromeframe]

[B56] **HTML5 Shiv** [https://code.google.com/p/html5shiv/]

[B57] **json2.js** [https://github.com/douglascrockford/JSON-js]

[B58] **Universal Sequence Maps** [http://usm.github.com/]

[B59] Zou Q Li XB Jiang WR Lin ZY Li GL Chen K **Survey of MapReduce frame operation in bioinformatics** Brief Bioinform 2013 [http://bib.oxfordjournals.org/content/early/2013/02/07/bib.bbs088.abstract] 10.1093/bib/bbs088 23396756

[B60] **LINPACK Benchmark** [http://www.top500.org/project/linpack/]

[B61] **Apache Hadoop** [https://hadoop.apache.org/]

[B62] **Web Workers** [http://www.w3.org/TR/workers/]

[B63] Tendulkar V Snyder R Pletcher J Butler K Shashidharan A Enck W **Abusing cloud-based browsers for fun and profit** Proceedings of the 28th Annual Computer Security Applications Conference, ACSAC ‘12 2012 New York: ACM 219 228 [http://doi.acm.org/10.1145/2420950.2420984]

[B64] Anderson DP Kubiatowicz J **The worldwide computer** Sci Am 2002 286 3 40 47 [March 2002 issue] 10.1038/scientificamerican0302-40 11857899

[B65] Karp AH **The global computer** Proceedings of the Fourth International Conference on Creating, Connecting and Collaborating through Computing, C5 ‘06 2006 Washington, DC: IEEE Computer Society 112 119 [http://dx.doi.org/10.1109/C5.2006.41]

[B66] **BLAST** [http://blast.ncbi.nlm.nih.gov/]

[B67] **The Perl Programming Language** [http://www.perl.org/]

[B68] **Python Programming Language** [http://www.python.org/]

[B69] **OAuth Community Site** [http://oauth.net/]

[B70] **Git distributed version control system** [http://git-scm.com/]

[B71] **Mercurial** [http://mercurial.selenic.com/]

